# Farm systems assessment of bioenergy feedstock production: Integrating bio-economic models and life cycle analysis approaches

**DOI:** 10.1016/j.agsy.2012.02.005

**Published:** 2012-06

**Authors:** N.J. Glithero, S.J. Ramsden, P. Wilson

**Affiliations:** Division of Agricultural and Environmental Sciences, School of Biosciences, University of Nottingham, Sutton Bonington Campus, LE12 5RD, United Kingdom

**Keywords:** Bioenergy, Cereal straw, Greenhouse gas emissions, Modelling, Farm systems

## Abstract

Climate change and energy security concerns have driven the development of policies that encourage bioenergy production. Meeting EU targets for the consumption of transport fuels from bioenergy by 2020 will require a large increase in the production of bioenergy feedstock. Initially an increase in ‘first generation’ biofuels was observed, however ‘food competition’ concerns have generated interest in second generation biofuels (SGBs). These SGBs can be produced from co-products (e.g. cereal straw) or energy crops (e.g. *miscanthus*), with the former largely negating food competition concerns. In order to assess the sustainability of feedstock supply for SGBs, the financial, environmental and energy costs and benefits of the farm system must be quantified. Previous research has captured financial costs and benefits through linear programming (LP) approaches, whilst environmental and energy metrics have been largely been undertaken within life cycle analysis (LCA) frameworks. Assessing aspects of the financial, environmental and energy sustainability of supplying co-product second generation biofuel (CPSGB) feedstocks at the farm level requires a framework that permits the trade-offs between these objectives to be quantified and understood. The development of a modelling framework for Managing Energy and Emissions Trade-Offs in Agriculture (MEETA Model) that combines bio-economic process modelling and LCA is presented together with input data parameters obtained from literature and industry sources. The MEETA model quantifies arable farm inputs and outputs in terms of financial, energy and emissions results. The model explicitly captures fertiliser: crop-yield relationships, plus the incorporation of straw or removal for sale, with associated nutrient impacts of incorporation/removal on the following crop in the rotation. Key results of crop-mix, machinery use, greenhouse gas (GHG) emissions per kg of crop product and energy use per hectare are in line with previous research and industry survey findings. Results show that the gross margin – energy trade-off is £36 GJ^−1^, representing the gross margin forgone by maximising net farm energy *cf.* maximising farm gross margin. The gross margin–GHG emission trade-off is £0.15 kg^−1^ CO_2_ eq, representing the gross margin forgone per kg of CO_2_ eq reduced when GHG emissions are minimised *cf.* maximising farm gross margin. The energy–GHG emission trade-off is 0.03 GJ kg^−1^ CO_2_ eq quantifying the reduction in net energy from the farm system per kg of CO_2_ eq reduced when minimising GHG emissions *cf.* maximising net farm energy. When both farm gross margin and net farm energy are maximised all the cereal straw is baled for sale. Sensitivity analysis of the model in relation to different prices of cereal straw shows that it becomes financially optimal to incorporate wheat straw at price of £11 t^−1^ for this co-product. Local market conditions for straw and farmer attitudes towards incorporation or sale of straw will impact on the straw price at which farmers will supply this potential bioenergy feedstock and represent important areas for future research.

## Highlights

► A modelling framework for managing energy and emissions trade-offs in agriculture. ► Combination of bio-economic process modelling and life cycle assessment. ► Captures financial, energy and emissions trade-offs and cereal straw use. ► The gross margin-energy trade-off is £36 GJ^−1^. ► All cereal straw is baled for sale when gross margin or net energy is maximised.

## 1 Introduction

Concerns relating to greenhouse gas (GHG) emissions and energy security have driven the development of policies that aim to substitute energy from biological systems that existed in the past (fossil energy) with those that exist in the present day (bioenergy). In the European Union, Directive 2009/28/EC sets a target of 10% of final consumption of energy in transport to be derived from renewable sources by 2020. This has increased demand for bioenergy in the form of biofuel; initially the increase has been met by ‘first generation’ fuels i.e. those that are derived from biological sources that can also serve as food supplies. However, this competition with food production, together with concerns over the small amount of surplus energy produced by some biofuel crops and the associated GHG emissions of growing and processing the crops, has led to an interest in ‘second generation biofuels’ (SGBs). These aim to negate some of the problems associated with first generation fuels by using agricultural ‘wastes’, such as cereal straw, as feedstocks for biofuels such as bio-ethanol. A useful distinction is to consider these as ‘co-product’ second generation biofuels (CPSGBs), contrasting with SGBs derived from dedicated energy crops (e.g. *miscanthus*).

There is much debate in the literature as to what agricultural sustainability entails and how it might be measured. From an economic perspective there are a number of factors that should be included in an assessment of the sustainability of biofuel production. First, what are the trade-offs between encouraging biofuels and other outputs of economic importance, particularly food crops? Second, what is the net energy value of potential feedstocks such as cereal straw; that is, the surplus energy potentially available for conversion to fuel after allowing for energy used in production of the feedstock? Third, what are the environmental effects of biofuels; in particular, what are their effects on GHG emissions given that part of the objective of encouraging biofuel production is to reduce GHGs? A further, more conceptually difficult consideration is the wider system effects of encouraging production of biofuel. In the case of cereal straw, these effects include the amount and timing of on-farm labour and machinery use, soil–straw interactions and fertiliser use and the extent to which farmers respond to policy-induced incentives – either through market prices or mechanisms such as subsidies and grants.

A common method of assessing the impact of biofuel production has been to use life cycle analysis (LCA). LCA is a procedure that attempts to account for the environmental effects of processes involved in the production of goods from ‘start to finish’. A framework for LCA is provided by the International Standards Organisation (ISO, Anon, 2006) although not all studies follow the full ISO recommendations. Elsayed et al. (2003) used LCA to create a set of energy and carbon balances for a range of energy products derived from biomass feedstocks including ‘food’ crops (oilseed rape, wheat, sugar beet), dedicated energy crops, and crop and forestry residues. A relatively early example of an energy balance calculation for biomass that examines first generation feedstocks including wheat and oilseed rape alongside residue feedstock (e.g. forestry residues) is given by Börjesson (1996). St. Clair et al. (2008) assess GHG emissions from *miscanthus*, short rotation coppice, wheat, oilseed rape and forestry feedstocks, whilst a much cited study by Kramer et al. (1999) addresses GHG emissions from conventional Dutch agriculture focusing on arable, root and field vegetable crops. Berry et al. (2010) consider disease management effects on GHG emissions from wheat production: when examined on a per tonne of grain basis, the authors found relatively little difference in GHG emissions between fungicide treated and untreated wheat when optimal levels of nitrogen fertiliser were applied. Edwards-Jones et al. (2009) calculate a ‘carbon footprint’ for different livestock production systems, highlighting the wide range of emissions values that can occur when estimating this metric depending on the system boundaries and the case study farms used. Some studies consider wider environmental impacts (Brentrup et al., 2004; Haas et al., 2000; Tuomisto et al., 2009; Williams et al., 2006) and an increasing number of papers consider energy and environmental impacts together (Cherubini and Ulgiati, 2010; Kaltschmitt et al., 1997; Pehnt, 2006). A criticism of LCA is that in its standard form, it fails to take account of changes in land use: thus, a process may have a relatively favourable environmental effect, as measured through LCA, but unfavourable production effects through foregone output – either food or other land based goods and services. Some studies using LCA consider land use change – for example, Berry et al. (2010) argue that maintaining current grain supplies in the absence of fungicide would require a far higher area of land to be used in wheat production, with consequent increases in GHG emissions, however land area change is not accounted for within the LCA itself.

Janssen and van Ittersum (2007) provide a review of ‘bio-economic’ models, citing many examples of models that attempt to capture trade-offs between agricultural production and profitability and a range of environmental variables, including nitrate loss, GHG emissions and biodiversity. However, relatively few of these models consider energy use and production. Gibbons et al. (2006) include the energy crop *miscanthus* in the *Farm-adapt* model; however, apart from fuel, no account is taken of other energy inputs into the farm system. A further advantage of bio-economic models that involve programming techniques (for example, linear programming and its variants) is that they account for the opportunity cost of resources other than land – labour and machinery. When environmental variables are introduced, the optimal solution to a programming model can be used as a guide to evaluating whether policies are sustainable in the sense that the optimal solution accounts for costs (e.g. resources no longer available for alternative uses) and benefits (e.g. net energy produced). More generally, bio-economic models are also an effective method for integrating biological and economic information.

This paper describes a bio-economic model designed as part of an inter-disciplinary research project funded by the UK’s Biotechnology and Biological Sciences Research Council (BBSRC). The focus of this project is to use cereal straw as an exemplar feedstock for lignocellulosic bioethanol production. The project is divided into the following elements: improving the efficiency of plant cell wall deconstruction; enhancing fermentation processes to bioethanol production; life cycle analyses; ‘societal and ethical reflections’; and a bio-economic farm model, ‘MEETA’ (Modelling Energy and Emissions Trade-offs in Agriculture). The objective of the MEETA model is to capture trade-offs between different aspects of the farm system – those that are not captured by the LCA analysis. However, MEETA also has an important conceptual role in demonstrating, within an inter-disciplinary framework, how a farm system producing cereal straw in the UK operates. For example, the MEETA model provides a framework for quantifying the change in nutrient and energy requirements from either incorporating cereal straw into the soil or baling and removing straw. Part of the sustainability assessment of cereal straw as a feedstock within the LACE project will depend on the farm level implications of its removal and the potential land use changes that can occur with changes in cereal and cereal straw prices.

The objectives of the paper are: (i) to describe the relevant components of a UK farm system growing cereal crops; (ii) to present a bio-economic model designed to capture trade-offs between net energy production, GHG emissions and profitability associated with this farm system; (iii) to measure these trade-offs by maximising/minimising different objectives within the MEETA model. The model is presented in its baseline form in Section 2, parameterised for a ‘mainly cereals’ farm typical of the eastern part of England. These farms grow a mix of ‘combinable’ crops, based around winter wheat, winter oilseed rape, winter and spring barley and winter beans. Baseline model results are presented in Section 3 and compared to available literature values, providing a validation phase; trade-offs between farm-level profitability, net energy and GHG emissions are quantified. A sensitivity analysis of the model to changes in cereal straw prices is also conducted. Section 4 provides concluding comments and avenues for future development of the MEETA model in the context of farmer decision making, alternative farm types and the inclusion of dedicated energy crops.

## 2 Materials and methods

The MEETA model is a linear programming optimisation model that represents multi-year cropping within a single year framework, based on combinable crops common to cereal farms in the UK. The main components of the model are: activities for crops, work rates for the various crop operations, levels of chemical applications (both in the form of crop protection products and fertilisers), initial seed requirements, grain drying requirements and the associated diesel use for this operation, yield data for grain and straw for each crop, contract costs and diesel use by machinery. Output from crops is a product of straw and grain at representative market values; the model maximises the gross margin between total output and variable costs of seeds, fertilisers and sprays, contract costs and fuel costs. Energy inputs and outputs and emissions data are associated with the main inputs and outputs using secondary data and LCA literature. The major constraints in the model are farm size, crop rotations and availability of on-farm machinery for some operations. The model can optimise for either (maximised) farm gross margin, net farm energy or (minimised) GHG emissions, and produces the optimal crop mix, associated machinery and contract use and the farm gross margin, net energy and GHG emissions for each optimal crop mix. Our objective for MEETA was to build a model that would improve understanding of the complexities of a typical arable farm system in the UK under conditions which include potential removal of straw as a lignocellulosic bioenergy feedstock. Transparency in defining model inputs and constraints was achieved by the initial construction of the model in Excel; the model will be transferred into GAMS (General Algebraic Modelling System) code as part of the ongoing inter-disciplinary project.

The model structure allows trade-offs between energy, emissions and financial performance to be quantified with a specific focus on bioenergy production. GHG emissions from agriculture (nitrous oxide, methane and carbon dioxide) are modelled individually (where data permit) and are directly related to the chemical inputs (e.g. fertiliser) applied and associated field operations (e.g. harvesting); these are both determined by the model, drawing upon land, machinery and labour constraints appropriate to the farm type. The quantification of energy used in the farm system and embodied in the farm outputs allow a ‘net energy’ (or ‘energy balance’) to be calculated. The MEETA model focuses on farm level trade-offs and hence the upper model boundary includes everything that is used on farm, up to the point of sale at the farm gate. The following sections provide more detail on the levels of complexity and boundaries for specific parts of the model.

### 2.1 Land and crops

The model was set up to represent a UK Cereal farm from the Farm Business Survey (the Farm Business Survey – FBS – is part of the Farm Accountancy Data Network) of 400 hectares (rounded average of 195 large Cereal farm areas in the FBS, 2007/2008). The crops available to the model are winter wheat (first wheat grown after a break crop, second wheat grown after a cereal, and continuous wheat), winter and spring barley, winter field beans and winter oilseed rape (the latter two crops are potential break crops i.e. are used to break up a cereal rotation). In 2011, cereals were grown on 67% of arable land in England and oilseed crops were grown on 18% (Anon, 2011a). Of the cereal area, winter wheat and barley were grown on 72% and 24% respectively and 91% of the oilseed crop area was in oilseed rape (Anon, 2011a). The most common break crop, other than oilseed rape, was field beans which has been included to give an alternative break crop. There are variations to some of the crops in the model to allow for crop rotation requirements, fertiliser management and straw removal for cereal crops. Winter wheat can have different levels of nitrogen fertiliser applied with associated levels of yield (see Section 2.3).

The model has a mixed rotational structure which contains two types of rotational constraint. The first type is a sequential constraint (Rae, 1994) that simulates both crop sequences over time (e.g. second winter wheat follows a first cereal) and differences in the supply and demand of nutrients over time (e.g. first winter wheat following winter beans requires less nitrogen than second winter wheat as there is assumed to be a greater residual supply of nitrogen in the soil). The sequential constraint also simulates differences in nutrient supply arising from cereal straw removal or incorporation from the previous crop after harvest. Thus cereals that follow cereals, where the straw has been removed, have different nutrient requirements to those that follow cereals where the straw was not removed. Nutrient supply and demand is calculated using the ‘RB209’ guidelines; see Section 2.3.

The second type of rotational constraint is proportional, and relates to crop areas in the model. A cereal production limit *λ* is used to restrict the proportion of crops that can follow cereals to being equal to or less than the proportion of first cereals i.e. those that follow a break crop. For example, when *λ* = 1 the area of crops that can follow cereals will be equal to or less than the area of first cereals (in this case with the other rotational constraints restricting the model to a 2-year cereal-break crop rotation) whereas when *λ* = 0.5 (the initial value used in this run of the model) the area of crops that can follow cereals will be equal to or less than double the area of first cereals. This constraint does not limit the length of the crop rotations that the model can find to be optimal but does restrict the areas of cropping. For example when *λ* = 0.5, 100% of the land can be attributed to either of the following rotations: cereal-break or cereal-cereal-break, since they both fulfil the proportion requirement. Alternatively, the land can have a mix of rotations: e.g. 50% each of cereal-break and three cereals followed by a break – this also satisfies the proportional constraint. Other proportional constraints are directly related to the nutrient requirements of the crops, e.g. the area of cereal crops following cereals where the straw has been removed has to be less than or equal to the area of cereal crops where the straw removal operation has occurred. The MEETA model is a static framework and gives a 1 year representation of a dynamic system and the constraints used are designed to represent this system. The advantage of this structure is that it does not restrict the model to rigid crop rotations. For example, agronomic advice to farmers for oilseed rape rotations would typically recommend that the crop be grown no more than once on the same land every 4 years; however, as noted by Ackrill et al. (2001), in practice, farmers grow oilseed rape more frequently than this. Rotations are therefore predominantly used to capture nutrient and yield relationships.

### 2.2 Machinery and labour use

Each crop in the model has specific machinery and labour requirements, in hours per hectare, based on required crop operations (e.g. ploughing, sowing) and their frequency (Table 1). The farm model has 2.8 full time workers (average from large Cereal farms in FBS energy component 2007/2008, including farmer and spouse supplied labour) that can operate the available machinery. Machinery available on the farm is shown in Table 2. Crop operations that require machinery not common to this type of farm can be bought in as contract machinery with associated labour (specifically the ‘swather’ for cutting oilseed rape and baler for straw). Each of the crop operations is designated as light, medium or heavy work and relevant tractor sizes are allocated to each work type. Machinery constraints restrict the on-farm use of machinery to equal to or less than the total number of workable hours available. If machinery hours above this are needed, contract machinery can be bought in; costs are shown in Table 2.

Direct and indirect energy and emissions for machinery use are captured in the model. The direct energy effects occur through the use of diesel which powers the machinery and is measured in mega joules (MJ) per litre (average of Wells, 2001; Woods and Bauen, 2003; Table 2). The price of diesel in the model is £0.6445 per litre (Anon, 2011b averaged over a 12 month period, November 2010 to October 2011). The emissions for diesel usage of nitrous oxide, carbon dioxide and methane are 0.7 g N_2_0 kg^−1^ diesel, 3.56 kg CO_2_ kg^−1^ diesel, 5.2 g CH_4_ kg^−1^ diesel respectively (Kramer et al., 1999). Diesel use and the associated direct energy for each of the machines are also shown in Table 2. The baseline combine harvester diesel use is defined when the straw is not chopped during the grain harvest: this leaves straw available ‘in the swath’ for baling. Diesel use when the straw is chopped on the combine is set at 20% more than the baseline fuel consumption (calculated from pers. comm. P. Freeman). The direct energy and emissions from grain drying and crop handling to and from the store are directly proportional to the yield of the crop (see Section 2.6).

Indirect energy and emissions are those embodied within the machinery during its manufacture. For the tractors and the combine harvester, values are taken from the Ecoinvent database (Anon, 2011c) embedded in the Simapro software. Emissions are calculated as a function of each machine’s weight (Table 2). The weight of the machines is calculated from the kW power of the tractors using the relationship between power and weight (Wells, 2001). The weight of the combine harvester is taken as the average of the two combines detailed in the Ecoinvent database. The weight for the baler (Wells, 2001) combined with Doering’s (1980) energy value was used to produce the embodied energy in the baler. The swather weight is calculated as one third of the average tractor weights (of the two medium-sized tractors) and is assumed to have the same indirect energy and emissions per kilogram as a tractor. Other machinery in the model are assumed to consist mainly of steel and are further assumed to have indirect energy and emissions data directly related to that used to produce steel. Tractors are assumed to have a 10,000 hour lifespan, whilst other machines, including combine harvesters, are assumed to have a 3000 h lifespan. These lifespans have been used to allocate the energy and emissions in the per-hour rates required when calculating the energy used per hectare of crop per year.

### 2.3 Fertilisers

Nutrient requirements for Nitrogen (N), Potash (K_2_O) and Phosphate (P_2_O_5_) differ between crops and are dependent on the previous crop grown (Table 3). Application levels were taken from RB209 (Anon, 2010) which is commonly used by the industry for nutrient management guidance. Over 80% of farmers and 90% of consultants indicated that RB209 has an influence of their fertiliser planning (Anon, 2008). In using these guidelines, we attempt to replicate what farmers do in practice, in the field. In addition, for the main straw-producing crop, winter wheat, N levels of 100%, 110%, 75% and 50% of the recommended amount are permitted crop choices within the model, with 110% allowing for farmer over application *cf*. the RB209 guidelines. Gross margin, net energy and GHG emission trade-offs can therefore be modelled for this part of the farm system. Application rates for K_2_O and P_2_O_5_ were taken from Anon (2009). These rates are increased, following RB209 guidelines, where straw is removed from the previous cereal crop. For winter oilseed rape there is no recommendation in the RB209 documentation for nutrient depletion relating to straw removal in previous cereal crops; we therefore make no adjustment to nutrient requirements for oilseed rape. The production of N, K_2_O and P_2_O_5_ releases GHG emissions and requires energy inputs (Table 4) which are included in the MEETA model. Release of nitrous oxide during the application of N fertiliser is an additional emission to that produced during the production of the fertiliser; we use Petersen et al.’s (2006) value of 1.6% of the N applied as the estimate of nitrous oxide release. The MEETA model also includes background emissions of soil released nitrous oxide of 1.4 kg N_2_O–N ha^−1^ yr^−1^ (Petersen et al., 2006).

### 2.4 Pesticides

Pesticide application data were taken from Garthwaite et al. (2006). Where products in the Garthwaite study are no longer commercially available it has been assumed that similar products have replaced them and that application rates are the same. The most common pesticides reported by Garthwaite et al. (2006), by area sprayed, are applied to each of the crops within the model; Table 5 shows the pesticide groupings used. Energy values for pesticide production were taken from Audsley et al. (2009); note that the Audsley study uses data from Green, 1987) which provides chemical specific energy values (Table 5).

The emissions associated with the production of pesticides are: carbon dioxide 3.96 kg CO_2_ kg^−1^ (Kramer et al., 1999), and methane 1.8∗10^−4^ kg CH_4_ kg^−1^ (Elsayed et al., 2003). Nitrous oxide emissions from pesticide production are assumed to be low (following Audsley et al., 2009) and are thus not included in the model. The cost per hectare for the pesticides applied to each crop (Table 5) are taken to be the average of the costs found in Anon (2011d), updated to October 2011 price levels (using Anon, 2011b). Where a cost of a pesticide application is unknown it has been taken to be the same as that of a cereal crop pesticide application, this only occurs once, in the case of seed treatments.

### 2.5 Other inputs to the crops

Each of the crops requires seed; application rates and prices (including royalty rates) were taken from Anon (2011d). The energy in the seed (MJ kg^−1^) is taken to be the energy production cost of the crop yield, as calculated by the model. For simplicity of application, this effectively represents a system where the farmer saves seed on-farm from one year to the next.

### 2.6 Crop outputs

Yields of grain and straw are shown in Table 6. As noted in Section 2.3, fertiliser use within the model for winter wheat can vary from the RB209 recommendation with attendant yield variations linked to changes in fertiliser level. To account for this the yield of grain for first winter wheat where the amount of N fertiliser applied differs from 100% of the RB209 recommendation is adjusted using the following function:

(1) $$y=11.228-6.346{\left(0.99\right)}^{x}-0.0104x$$

where *y* is yield and *x* is the application of N fertiliser applied. This functional form was developed for winter wheat by Sylvester-Bradley et al. (1984) and has been widely used since. The yield of straw for these reduced N crops is assumed to be proportional to the grain yield. For the second and continuous winter wheat crops the yield produced by (1) is reduced by 10% (Anon, 2011d). The grain for each of the crops is assumed to need drying before sale to reduce the moisture content by 3% to a level of 15%. Following the methodology of the FBS research programme, this requires 65 MJ of energy per 1% moisture content removed which is assumed to be provided by diesel fuel. The cost of grain drying is therefore based on the grain yield of the crop and the price of diesel. The emissions from the grain drying process are due to the consumption of the diesel fuel.

### 2.7 Time constraints

The model operates on three distinct labour time constraints which are based on the intensity of labour and machinery operations in the arable cropping year in the UK. The periods are peak harvest (mid-August to the end of the first quarter of September), peak cultivation (2nd quarter of September to the end of October) and the remainder of the year (excluding December and January during which almost no in field arable operations occur). The ‘remainder of the year’ constraint includes all operations for crops that occur over this period. Production on the representative farm type is dominated by winter crops; however, a more detailed representation of potentially binding constraints in spring would be required if an increase in spring cropping was anticipated. The approach follows Gibbons et al. (2010) where, in tests for model parsimony, weekly time-constraints were shown to be redundant in a substantial number of model runs.

## 3 Results and discussion

### 3.1 Model results

Results for the maximised farm-level gross margin, the maximised net farm energy and the minimised GHG emissions are shown in Table 7. When maximising the gross margin, the crop mix is 33.3% each of first winter wheat (where 75% of the RB209 recommendation of N fertiliser is applied and the cereal straw is baled), winter barley (where the cereal straw is baled) and winter oilseed rape. The total gross margin, net farm energy and GHG emissions for this solution are £714 ha^−1^, 64 GJ ha^−1^, 4432 kg CO_2_ eq ha^−1^ respectively. When maximising the net farm energy the crop mix is 50% each of winter wheat (where 75% of the RB209 recommendation of N fertiliser is applied and the cereal straw is baled) and winter field beans. The total gross margin, net farm energy and GHG emissions for this solution are £672 ha^−1^, 65 GJ ha^−1^ and 2335 kg CO_2_ eq ha^−1^ respectively. When minimising the GHG emissions the crop mix is 50% each of winter wheat (where 50% of the RB209 recommendation of N fertiliser is applied and the cereal straw is not baled) and winter field beans. The total gross margin, net farm energy and GHG emissions for this solution are £605 ha^−1^, 52 GJ ha^−1^ and 1903 kg CO_2_ eq ha^−1^ respectively.

The solution when the net farm energy is maximised is similar to the optimised gross margin solution, in terms of both net energy and baling of cereal straw (at a wheat straw price of £43 t^−1^ all cereal straw is baled in both cases); however, the gross margin is 6% and GHG emissions 47% less (Table 7) than the maximised gross margin solution. Minimising the farm GHG emissions rather than maximising the farm gross margin produces a 57% reduction in GHG emissions and decreases the gross margin and net farm energy by 15% and 19% respectively. The trade-offs are the marginal changes between the optimised solutions rather than the costs of production *per se*; they thus give an indication of the financial incentive required to change production based on the profit maximising objective assumed in MEETA. On this reasoning, the gross margin-energy trade-off has a value of £36 GJ^−1^ which represents the gross margin forgone per GJ of additional net energy produced by comparing the outputs from the two contrasting model solutions (i.e. gross margin and net energy maximisation). The gross margin-GHG emission trade-off is £0.15 kg^−1^ CO_2_ eq and the energy-GHG emission trade-off is 0.03 GJ kg^−1^ CO_2_ eq which represent the gross margin or net energy forgone per kg of CO_2_ eq emissions saved when comparing the gross margin and net energy model solutions against the GHG emission solution.

The crop mix proportions were compared to data from the Farm Business Survey 2010/2011. As noted, when the gross margin is maximised the crop proportions are one-third each of first winter wheat, winter oilseed rape and winter barley. The average proportions of these crops in the FBS data, with crops restricted to those present in the model, are 58.9% winter wheat, 8% winter barley and 22.3% winter oilseed rape. Authors’ calculations from FBS data for England 2007/2008 (most recent year for which this data exists) suggest that cereal straw is baled on approximately half of the cereals’ area on Cereal farms. All cereal straw is baled in the MEETA model when gross margin is maximised; however, straw prices were lower over this period (wheat straw in ‘big square bales’ was £26–31 t^−1^ over 2007–2008, Anon, 2011e). Model sensitivity to changes in cereal straw prices is investigated in Section 3.2.

GHG emissions of individual crops have been calculated as kg of CO_2_ eq released per kg of crop grain. Table 8 shows values with emissions from soils included and excluded allowing comparison with existing literature values which have different system assumptions and boundaries; all values are calculated from the optimised gross margin model results, with the exception of winter field beans which is calculated when the net farm energy is maximised. Williams et al.’s (2006) study specifically relates to bread wheat which in part explains why this value is greater than other studies for the winter wheat emissions. Just over half of the nitrous oxide emissions for winter wheat and winter barley (54% and 56% respectively) flow from the N fertiliser when the gross margin is maximised, these results are similar to the findings of Kramer et al. (1999).

**Fig. 1:**
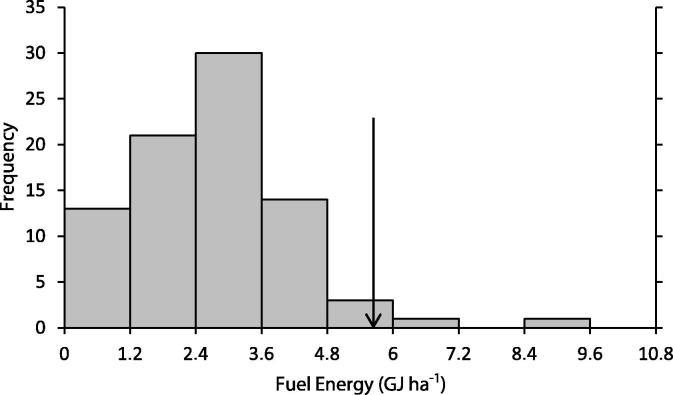
Histogram of fuel energy per ha for FBS Cereal farms (GJ ha^−1^). The arrow represents the value of the fuel energy per ha for on farm machinery from the MEETA model.

**Fig. 2:**
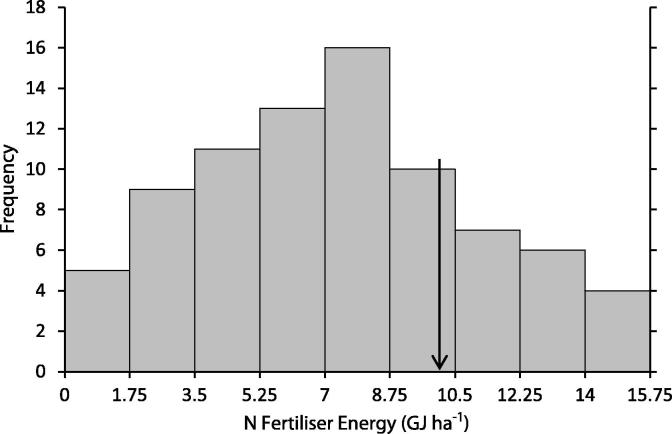
Histogram of nitrogen fertiliser energy per ha for FBS Cereal farms (two outliers in the dataset removed) (GJ ha^−1^). The arrow represents the value of the nitrogen fertiliser energy per ha from the MEETA model.

The MEETA model results when the farm gross margin is maximised were also compared with survey data (FBS energy survey, conducted in 2007/2008). In the survey, energy use per hectare from diesel ranges from 0 to 9.438 GJ ha^−1^ (diesel use divided by the utilised agricultural area, Fig. 1): this excludes energy from contracted machinery use; hence, some farms within the survey report a zero value for own diesel use. Energy associated with machinery fuel use from this run of the MEETA model is 5.79 GJ ha^−1^, excluding contracted machinery fuel use. The model result with respect to diesel use is towards the upper value of the FBS energy distribution, reflecting the lack of contract energy use data in the survey. The FBS energy survey range for the energy per hectare from N fertiliser use is 0–15.47 GJ ha^−1^ (Fig. 2, N use divided by the utilised agricultural area; zero values reflect organic farms). The MEETA model N fertiliser energy is 10.42 GJ ha^−1^.

**Fig. 3:**
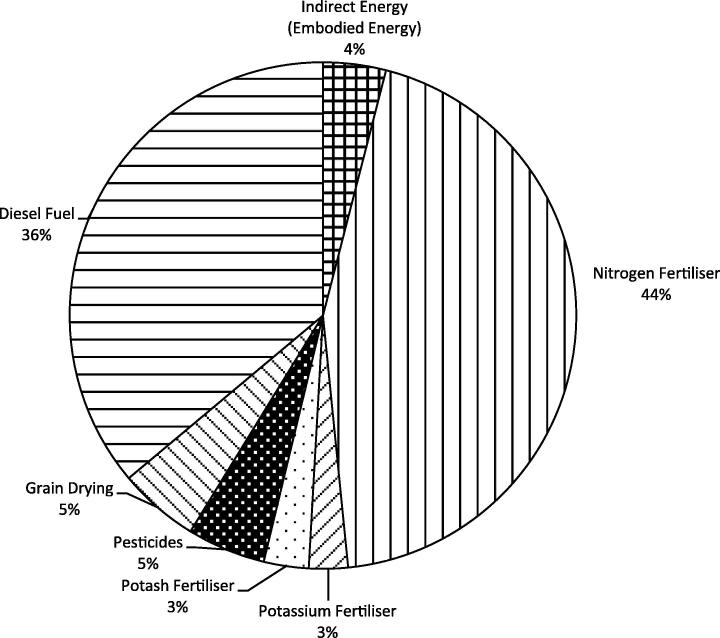
Energy inputs into the farm system (optimised for gross margin) as a representation of the total amount of energy used.

Fig. 3 shows the relative contributions of the different energy inputs to the total energy applied to the farm on a per hectare basis. As expected, N fertiliser and diesel fuel are the greatest contributors to the energy used on farm, accounting for 80% of total energy use. The importance of N fertiliser (indirect energy) and diesel fuel (direct energy) to the overall energy input at the farm level is well documented. Meul et al. (2007) report that diesel fuel and mineral fertilisers represent 36% and 38% of the energy inputs to specialised arable farms in Flanders. With respect to mineral fertilisers only, Nguyen and Haynes (1995), in a study of mixed cropping farms in New Zealand, cite a range of 23–63% (mean of 45%) of total energy inputs for cereal crop production.

### 3.2 Straw price sensitivity analysis

**Fig. 4:**
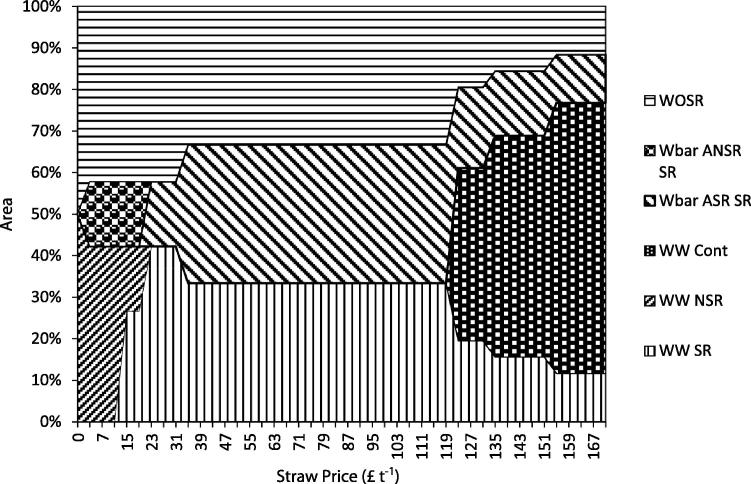
Crop mixes under varying cereal straw prices. ASR – after a cereal crop where straw was removed. ANSR – after a cereal crop where no straw removed. NSR – no straw removed. SR – straw removed. WW – winter wheat. Wbar – winter barley. WOSR – winter oilseed rape. Cont – continuous.

To test the sensitivity of the model to changes in the input parameter values, the price of cereal straw, both wheat and barley, was varied, other things held equal, to assess model outputs with respect to baling, nutrient use and crop mix. The model price for wheat straw (£43 t^−1^) was varied within the range of £0–171 t^−1^. This is a larger range than has been historically the case – the price for large straw bales from 2000 to 2010 ranged from £14.75 t^−1^ to £53 t^−1^ (Anon, 2011e). However, the upper value takes the wheat straw price to a level comparable with recent (2008–2011) wheat grain prices. Barley straw price tracks the price of wheat straw with the former valued at £16 t^−1^ more than the latter (average of a single years’ price data, Anon, 2011e); When maximising the net farm energy the model will always bale cereal straw regardless of the price (even if it has no monetary value). When minimising the GHG emissions the model will not bale the straw regardless of the price. Therefore, the model was run to maximise the farm gross margin at different cereal straw price levels. The crop mix changes are shown in Fig. 4; when the cereal straw price is high relative to crop prices, the cropping mix moves towards continuous winter wheat. As expected, as the price of cereal straw falls, the model moves to a crop mix where the straw is not baled as it no longer becomes economically viable to do so. The results reflect the profit maximising assumptions built into MEETA and do not take account of other factors which may influence straw use decisions. For example, some farmers may prefer to chop and incorporate straw to avoid timeliness penalties resulting from the use of contract services. Baling and removal of straw increases the amount of farm machinery movement on the land which can increase soil compaction.

**Fig. 5:**
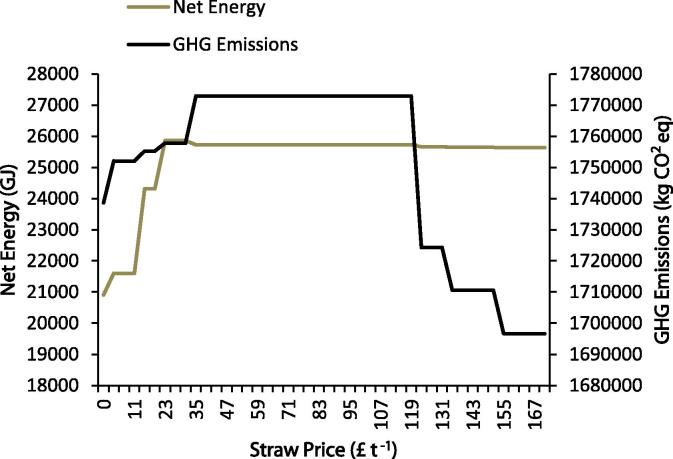
Net farm energy and GHG emissions under varying cereal straw prices.

The net energy and GHG emissions for these changes in the cereal straw price can be seen in Fig. 5. GHG emissions range between *circa* 1,696,700 and 1,772,900 kg CO_2_ eq and the net energy varies between 20,903 and 25,866 GJ. The GHG emission impact of removing straw is relatively small in comparison to that associated with producing the crops themselves: an increase of 5733 kg CO_2_ eq or 0.3% more compared to the equivalent rotation where the straw is removed. The additional external P_2_O_5_ and K_2_O requirements when straw is removed, together with additional fuel and embodied energy associated with baling, is offset by the fuel requirements associated with chopping the straw as part of the combining activities. The net effect is a small increase in emissions when the straw is baled. It is worth noting that P_2_O_5_ and K_2_O fertiliser have lower global warming potential in comparison to N fertiliser: following the RB209 guidelines, the MEETA model assumes that only non-N nutrients are affected by straw removal. Thus, overall, while net energy impacts of straw removal are relatively large, GHG impacts of straw removal compared with straw incorporation are relatively modest.

### 3.3 Crop area constraint sensitivity analysis

Both the GHG minimisation and net energy maximisation runs lead to substantial increases in the area of winter beans. To examine the impact of restricting the model to a more typical mix of break crops for this farm type, the area of winter field beans was restricted to a maximum of 10% of the farm area and oilseed rape was restricted to only follow cereal crops. Maximising the gross margin with these further constraints has no effect on the solution described previously, the crop mix remains 33.3% each of first winter wheat (where 75% of the RB209 recommendation of N fertiliser is applied and the cereal straw is baled), winter barley (where the cereal straw is baled) and winter oilseed rape. When maximising net energy, these additional constraints change the crop mix to 50%, 10% and 40% of first winter wheat (where 75% of the RB209 recommendation of N fertiliser is applied and the cereal straw is baled), winter field beans and winter oilseed rape respectively. The total gross margin, net farm energy and GHG emissions for this solution are £681 ha^−1^, 65 GJ ha^−1^, 3954 kg CO_2_ eq ha^−1^ respectively. When minimising the GHG emissions with these additional constraints the crop mix becomes 10% of first winter wheat (where 50% of the RB209 recommendation of N fertiliser is applied and the cereal straw is not baled), 10% of second winter wheat which follows an un-baled cereal crop (where 50% of the RB209 recommendation of N fertiliser is applied and the cereal straw is not baled), 10% of winter field beans and 70% of continuous winter wheat (where 50% of the RB209 recommendation of N fertiliser is applied and the cereal straw is not baled) respectively. The total gross margin, net farm energy and GHG emissions for this solution are £564 ha^−1^, 44 GJ ha^−1^ and 2902 kg CO_2_ eq ha^−1^ respectively.

The maximised net energy produced with the additional constraints is 0.64% less than the unconstrained solution and the gross margin-energy trade-off has a higher value of £44 GJ^−1^ which represents the gross margin forgone per GJ of additional net energy produced by comparing the outputs from the two contrasting model solutions (i.e. gross margin and net energy (with further constraints maximisation)). The minimised GHG emissions produced with the additional constraints are 52% higher than the unconstrained solution and the gross margin-energy trade-off has a lower value of £0.11 kg^−1^ CO_2_ eq.

The additional constraints have no effect on the gross margin solution; this is expected since the original crop mix solution does not contain any winter field beans. Maximising the net farm energy with these additional constraints leads to a different break crop mix but does not alter the overall ratio of cereal to break crop or the cereal crop (wheat) used in the solution. The GHG emissions solution with the additional constraints is different to the original solution since the model grows as much winter field beans as possible and then grows winter wheat on the rest of the available farm land, this is due to the higher GHG emissions associated with winter oilseed rape in comparison to the 50% N fertiliser winter wheat (uses 50% of the RB209 recommendation) which is caused by the difference in N fertiliser applied to the two crops.

### 3.4 MEETA model in the context of farm decision making

The MEETA model provides financial, net energy and GHG emissions metrics with functionality to optimise for each of these objectives. This approach builds upon an established tradition of using mathematical optimisation programming to examine farm-level system impacts, in particular examining financially optimal crop mix and associated activities and inputs. However, the MEETA makes no allowance for farmer objectives beyond profit maximisation, and in particular differing attitudes and drivers behind farmer decision making with respect to straw use are not captured within the model. Hence, whilst the MEETA model provides a framework for examining a representative farm-system within a quantitative analysis, it does not provide a predictive tool for assessing crop mix and straw use decisions *per se* in the absence of, respectively, farm and farmer geographical and attitudinal data. For example, within the Eastern parts of East Anglia, straw sale possibilities are currently considerably more constrained than within more central areas of England due to the high relative transport costs of moving straw for use to more Western areas. Straw use decisions are additionally influenced by farmer attitudes towards soil compaction and structure, timeliness of crop establishment following harvest and attitudes towards managing further crop operations (e.g. baling and straw carting) during the harvest period. Understanding the physical output, farm financial, net energy and GHG consequences of different cropping patterns and straw use lies at the heart of feedstock sustainability assessment within the LACE programme. In order to provide a full assessment of crop mix and straw use decisions under market and policy scenarios a more holistic approach will be required combining the approach of the MEETA model with data on farmer attitudes and behaviours.

### 3.5 Further development of the MEETA model

In building the MEETA model, we have used data, for example, from the Ecoinvent database that would be used in an LCA analysis. MEETA therefore complements the LCA approach and allows land use and other resource use impacts to be assessed. The approach has also allowed new data and approaches (FBS fuel use, RB209 guidelines) to be incorporated into the model. The reliance on secondary data has disadvantages when compared to using linked modelling approaches: we only include one nitrogen response function – albeit for the main grain and straw-producing crop, winter wheat – and GHGs, other than those linked to this response function, are obtained from the literature. However, the process of adding parameters to a model can also lead to problems of poor predictive performance (Cox et al., 2006). In our case it would also have taken MEETA beyond the modified LCA approach that was required within the linked themes of the LACE project. The RB209 guidelines are useful to reflect farm practice relating to the level of nutrients applied to crops, in England, but less useful in providing information on the effects of these farm practices, particularly where straw is removed or not removed. Whilst the MEETA model could be developed further, for example to include recent information on the effects of straw removal on soil properties (e.g. Powlson et al., 2011) and to include other environmental variables such as nitrate loss and, following Tzilivakis et al. (2005) measures of biodiversity impact, problems of uncertainty of response remain. This uncertainty exists both in terms of the farmer decision making outlined above and the interactions between plant, soil and environment. Hence, further development of the MEETA model to include such interactions would prove fruitful in attempting to more completely understand the farm system.

## 4 Conclusion

The results of the MEETA model under contemporary agricultural practices and market conditions generates machinery use and crop areas that are in line with current English cropping patterns and diesel and fertiliser use on cereal farms. The MEETA model therefore provides an adequate and appropriate representation of an arable farming system. Because the model contains financial, energy input requirements, total system energy output and emissions information, it can be used to investigate trade-offs that occur when different objectives are specified. The cases explored in this paper are the difference between maximising gross margin, maximising net energy and minimising GHG emissions. These outputs provide insight into the complex implications of CPSGB feedstock production if farms are required to maximise net energy rather than maximise financial returns. The MEETA model thus combines aspects of finance (gross margin), production (crop areas and yields) GHG emissions and energy balances associated with a particular agricultural system. It is a framework that can be extended to incorporate other farm types and other crops (e.g. *miscanthus*) without substantially increasing model complexity or reducing its transparency. It explicitly models the possibility of straw removal and its rotational nutrient consequences according to Anon (2010) which makes it an effective tool for assessing some aspects of farm level land use and sustainability that may be affected by governmental policies relating to SGBs from co-products such as cereal straw. In order to provide a more holistic framework for assessing the potential for CPSGB feedstock using cereal straw, further research should seek to understand farmer attitudes towards straw use, in particular analysing these attitudes in relation to farm type, location and managerial biographical factors and behaviours. This approach will overcome some of the limitations in both LCA and ‘systems’ modelling and allows substantial scope for further developing the two techniques. Second generation biofuels represent a potential source of future energy supply. Farm-level sustainability assessment is required as a necessary condition to understanding bioenergy sustainability using these advanced technologies.

## Figures and Tables

**Table 1:** Work rates for field operations and frequency of each of these operations for each crop. Work rates taken from Anon (2011d). The number of operations applied to each crop is taken from Nix (2010) and expert advice. The work rates for winter wheat are shown for 1st wheat where 100% of the recommended nitrogen fertiliser is applied.

	Field operation	hr ha^−1^	hr t^−1^	Winter wheat (1st wheat, 100% *N*)	Winter oilseed rape	Winter barley	Spring barley	Winter field beans
Cultivations	Plough (6 furrow) – heavy land	1.18		1	1	1	1	1
	Power harrow 4 m – heavy land	1.11		2	2	2	1	0

Drilling/seeding	Precision Drill 12 row	0.71		1	1	1	1	1

Crop maintenance	Spraying 24 m	0.14		3	4	3	2	2
	Fertilising – spinning	0.17		3	3	2	2	1

Crop harvest	Combining 6 m winter cereals	0.69		1	0	1	0	0
	Combining 6 m spring cereals	0.59		0	0	0	1	0
	Combining 6 m oilseeds (direct)	0.83		0	1	0	0	0
	Combining 6 m pulses	0.63		0	0	0	0	1
	Swather 4 m	0.61		0	1	0	0	0
	Grain cart (two trailer, one tractor)	0.83		1	1	1	1	1
	Straw baling (big round bales)	0.50		1	0	1	1	0
	Straw carting (two men tractor loader and trailers)	0.50		1	0	1	1	0
	Crop handling to/from store		0.03	0.25	0.099	0.21	0.16	0.12
	Drying (manned cont flow dryer)		0.07	0.58	0.231	0.49	0.37	0.28

**Table 2:** Farm machinery weights, diesel use, contract costs, direct and indirect energy and emissions. The weights of the tractors are calculated from Wells (2001), other machinery weights are taken from industry sources. The diesel usage is calculated from Anon (2001). The emissions, both direct and indirect, are calculated from the diesel use and the weights of the machinery. Contract costs are calculated from Anon (2001).

Machines	Weight (kg)	Diesel use (l h^−1^)	Direct energy (GJ h^−1^)	Indirect energy (MJ h^−1^)	Direct emissions (kg CO_2_ eq h^−1^)	Indirect emissions (kg CO_2_ eq h^−1^)	Contract cost (£ h^−1^)
Tractor <75 kW^a^	2473	9.90	0.36	34.06	32.09	1.76	31.39
Tractor 75 < 150 kW^a^	4756	22.60	0.83	65.51	73.25	3.39	50.38
Tractor 150 < 250 kW^a^	7799	40.20	1.48	107.41	130.29	5.55	76.12
Tractor > 250 kW^a^	8378	56.50	2.08	115.41	183.12	5.96	89.36
Plough	1950			14.86		1.01	
Subsoiler	5500			42.17		2.86	
Power harrow	990			7.59		0.51	
Drill	7400			56.73		3.85	
Sprayer	1120			8.59		0.58	
Trailer	3760			86.48		5.87	
Combine harvester	8250	34.85^b^	1.28^b^	265.07	112.95	15.32	143.46^c^
Baler	2000			53.33		4.80	45.63
Swather	2100	18.11	0.67	96.43	58.70	4.98	68.67
Grain dryer	3400			26.07		1.77	

a The upper limit of the tractor ranges is used when calculating the weight except for the >250 kW tractor where a 269 kW tractor was used as the calculation point.

b This is the diesel usage and direct energy where the straw is not chopped by the combine harvester.

c This is the contract fee for when the straw is not chopped by the combine harvester. If the straw is chopped then this is increased by the cost of the extra diesel needed to perform straw chopping.

**Table 3:** Fertiliser requirements for the crops within the MEETA Model calculated from Anon (2010) and Anon (2009).

Crop	N (kg ha^−1^)	P_2_O_5_ (kg ha^−1^)	K_2_O (kg ha^−1^)
1st Winter wheat	190	60	74
2nd Winter wheat following a cereal which had no straw removed	220	60	74
2nd Winter wheat following a cereal which had its straw removed	220	70	124
Spring barley following a break crop (e.g. winter field beans or winter oilseed rape)	110	46	63
Spring barley following a cereal which had no straw removed	140	46	63
Spring barley following a cereal which had its straw removed	140	56	108
Winter barley following a break crop (e.g. winter field beans or winter oilseed rape)	150	54	73
Winter barley following a cereal which had no straw removed	190	54	73
Winter barley following a cereal which had its straw removed	190	64	123
Winter field beans	0	60	64
Oilseed rape following cereal	220	61	70
Oilseed rape following winter field beans	190	61	70

**Table 4:** Prices, emissions and energy used in the production of fertilisers.

	Price (£ t^−1^)^b^	Energy (MJ kg^−1^)	Emissions (kg kg^−1^)			
			CO_2_	N_2_O	CH_4_	CO_2_ eq^a^
N	939.36	56.58^c^	2.66^d^	3.05E−2^d^	1.74E−3^d^	11.79
P_2_O_5_	398.39	9.45^f^	2.23E−1^d^	4.20E−5^e^	2.30E−5^e^	2.36E−1
K_2_O	469.64	7.55^f^	1.63E−1^d^	9.40E−5^e^	2.10E−5^e^	1.92E−1

a The global warming potential (GWP) factors (100 yr timescale) (Solomon et al., 2007) for the gases are used to give the emissions in CO_2_ eq (N_2_O and CH_4_ give a GWP 298 and 25 times greater respectively than CO_2_).

b The prices for the fertilisers are calculated from Anon (2011b) as a 12 month average from November 2010 to October 2011.

c N energy is the mean of the range of values found in: Wells (2001), Mortimer et al. (2003), Elsayed et al. (2003).

d Value/s from Kramer et al. (1999).

e Value/s from Elsayed et al. (2003).

f P_2_O_5_ and K_2_O energies are the mean of values found in Elsayed et al. (2003) and Anon (2009).

**Table 5:** Number of pesticides, applied to each of the crops (Garthwaite et al., 2006) and the energy (MJ) applied per hectare to each of the crops though the use of pesticides and their overall cost (costs are the authors calculations based on the prices given in Anon (2011d)) in £ per hectare.

		Winter wheat	Winter barley	Spring barley	Winter oilseed rape	Winter field beans
Fungicides	Chemicals	3	2	2	2	2
	Cost	68.95	45.97	45.97	29.14	37.01
	Energy	420	305	203	102	282

Herbicides	Chemicals	3	2	2	3	2
	Cost	36.01	24.01	24.01	89.43	64.93
	Energy	623	778	130	876	588

Growth regulators	Chemicals	2	1	0	0	0
	Cost	22.54	11.27	–	–	–
	Energy	397	295	–	–	–

Insecticides	Chemical	1	1	0	2	2
	Cost	5.80	5.80	–	12.87	12.87
	Energy	17	17	–	21	18

Seed treatments^a^ and mollusicides	Chemical	1	1	1	2	0
	Cost	14.19 (16.09)	13.72	15.61	20.66	–

Seed treatment and mollusicides combined	Energy	5	6	7	7	–
Total cost		147.50 (149.39)	100.77	85.59	152.10	114.81
Total energy		1462	1401	340	1006	888

a The cost of the winter wheat seed treatment has two values the first is for first winter wheat and the one in brackets for second and continuous winter wheat.

**Table 6:** Crop yields, where 100% of the RB209 (Anon, 2010) recommended amount of fertiliser is applied, prices and energies.

Crop	Grain yield^a^ (t ha^−1^)	Reference	Energy^b^ (GJ t^−1^)	Reference	Grain price^c^ (£ t^−1^)	Straw yield^a^ (t ha^−1^)	Reference	Energy^b^ (GJ t^−1^)	Reference	Straw price^d^ (£ t^−1^)
Winter wheat 1st	8.3	HGCA data, Lang and Allin (2006), FBS data (2004–2008), Nix (2008), Anon (2011d)	8.82^e^	Elsayed et al. (2003)	172.36	3.5	Nix (2008)	7.44	*Elsayed et al. (2003)*	43
Spring barley	5.4	Lang and Allin (2006), FBS data (2004–2006), Anon (2011d)	8.82	Assumed to be the same as for winter wheat	164.42	3	Anon (2011d)	7.44	*Assumed to be the same as for winter wheat*	59
Winter barley	7.0	HGCA data, Lang and Allin (2006), FBS data (2004–2006), Nix (2008), Anon (2011d)	8.82	Assumed to be the same as for winter wheat	164.42	3.6	Nix (2008), Anon (2011d)	7.44	*Assumed to be the same as for winter wheat*	59
Winter field beans	4.0	Nix (2008), Anon (2011d)	16.42	Moerschner and Lücke (2002)	206.67	0.0		0		0
Winter oilseed rape	3.3	FBS data (2004–2008), Nix (2008), Anon (2011d)	23.95^e^	Elsayed et al. (2003)	374.08	0		0		0

a Crop yield values are averages taken from various literature sources, shown to two significant figures.

b The energies of the grain and straw are shown for each crop shown to two decimal places.

c The prices of the grain yields are taken from Anon (2011b) averaged from November 2010 to October 2011. The barley grain price is fixed to be £7.94 t^−1^ less than the wheat grain price as this is the calculated difference between these two prices over the time period noted.

d The prices of the straw are taken from Anon (2011b) and from Defra (Anon, 2011e). The barley straw price is fixed to be £16 t^−1^ more than the wheat straw price as this is the calculated difference between these two prices over the time period noted.

e Energy value for the conversion of the wheat grain into bioethanol and for the conversion of oilseed rape into biodiesel.

**Table 7:** Baseline results of the MEETA model.

		Gross margin maximised	Net energy maximised	GHG emissions minimised
Crop Mix^a^	Winter wheat (SR, 75% N)	133.33	200	0
	Winter wheat (NSR, 50% N)	0	0	200
	Winter barley (ASR, SR)	133.33	0	0
	Winter field beans	0	200	200
	Winter oilseed rape	133.33	0	0

Finance	Overall farm costs	263,284	197,567	179,446
	Overall farm revenue	549,066	466,238	421,519
	Gross margin	285,782	268,671	242,072

Energy	In	9367	5752	5090
	Out	35,115	31,952	26,033
	Net	25,727	26,200	20,942

GHG emissions		1,772,947	933,841	761,354

a SR – straw removed, 75% N where 75% of the recommended nitrogen fertiliser has been applied, NSR – no straw is removed, 50% N where 50% of the recommended nitrogen fertiliser has been applied, ASR – crop is grown after a cereal crop where the straw was removed.

**Table 8:** Comparison of model results for the GHG emissions (in kg CO_2_ eq kg^−1^ grain) associated with each of the crops to literature values when nitrous oxide emissions from soil are/are not included.

		Model value	Literature value	Reference
Winter wheat	With soil emissions	0.457	0.804	Williams et al. (2006)^a^
			0.417	Berry et al. (2010)
	Without soil emissions	0.324	0.399	Kramer et al. (1999)
Winter barley	Without soil emissions	0.463	0.326	Kramer et al. (1999)
Winter oilseed rape	With soil emissions	1.50	1.71	Williams et al. (2006)
Winter field beans	With soil emissions	0.227^b^		

a Specifically relates to a bread wheat variety.

b This value is calculated when the net farm energy is maximised.
